# Supplementary material for: End Sequence Analysis Toolkit (ESAT) expands the extractable information from single-cell RNA-seq data
Source: Genome Res. 2016 Oct;26(10):1397–410. doi: 10.1101/gr.207902.116 (PMC5052061; doi:10.1101/gr.207902.116)
Supplement: Supplemental Material [file supp_gr.207902.116_Supp_Materials.tar.gz › ESAT-esat_v0.1/doc/html/classbroad_1_1pda_1_1geneexpression_1_1_pa_g_e_java.html]

ESAT: broad.pda.geneexpression.PaGEJava Class Reference


ESAT


- Main Page
- Related Pages
- Packages
- Classes
- Files

- Class List
- Class Index
- Class Hierarchy
- Class Members

All Classes Namespaces Files Functions Variables Enumerator Pages

- broad
- pda
- geneexpression
- PaGEJava

Public Member Functions |
Static Public Member Functions |
List of all members

broad.pda.geneexpression.PaGEJava Class Reference

Collaboration diagram for broad.pda.geneexpression.PaGEJava:

[legend]

|  |  |
| --- | --- |
| Public Member Functions | |
|  | PaGEJava (MatrixWithHeaders expression, Map< String, ArrayList > groupIndexes, String controlGroup) |
|  | |
|  | PaGEJava (File gctFile, File clsFile) throws Exception |
|  | |
| Map< String, Collection< String > > | getSignificantGeneSets (double alpha, double minFold) |
|  | |
| void | write (String save, File chipfile, double alpha, double fold) throws IOException |
|  | |

|  |  |
| --- | --- |
| Static Public Member Functions | |
| static void | main (String[] args) throws Exception |
|  | |

## Constructor & Destructor Documentation

|  |  |  |  |
| --- | --- | --- | --- |
| broad.pda.geneexpression.PaGEJava.PaGEJava | ( | MatrixWithHeaders | *expression*, |
|  |  | Map< String, ArrayList > | *groupIndexes*, |
|  |  | String | *controlGroup* |
|  | ) |  |  |

Here is the caller graph for this function:

|  |  |  |  |
| --- | --- | --- | --- |
| broad.pda.geneexpression.PaGEJava.PaGEJava | ( | File | *gctFile*, |
|  |  | File | *clsFile* |
|  | ) |  | throws Exception |

## Member Function Documentation

|  |  |  |  |
| --- | --- | --- | --- |
| Map<String, Collection<String> > broad.pda.geneexpression.PaGEJava.getSignificantGeneSets | ( | double | *alpha*, |
|  |  | double | *minFold* |
|  | ) |  |  |

Here is the call graph for this function:

|  |  |  |  |  |  |
| --- | --- | --- | --- | --- | --- |
| static void broad.pda.geneexpression.PaGEJava.main | ( | String[] | *args* | ) | throws Exception |

static

Here is the call graph for this function:

|  |  |  |  |
| --- | --- | --- | --- |
| void broad.pda.geneexpression.PaGEJava.write | ( | String | *save*, |
|  |  | File | *chipfile*, |
|  |  | double | *alpha*, |
|  |  | double | *fold* |
|  | ) |  | throws IOException |

Here is the call graph for this function:

---

The documentation for this class was generated from the following file:

- java/broad/pda/geneexpression/PaGEJava.java


---

Generated on Fri Aug 15 2014 15:11:00 for ESAT by  

 1.8.7
